# Meningeal defects alter the tangential migration of cortical interneurons in *Foxc1^hith/hith ^*mice

**DOI:** 10.1186/1749-8104-7-2

**Published:** 2012-01-17

**Authors:** Konstantinos Zarbalis, Youngshik Choe, Julie A Siegenthaler, Lori A Orosco, Samuel J Pleasure

**Affiliations:** 1 Institute of Pediatric Regenerative Medicine Shriners Hospitals for Children, Northern California, 2425 Stockton Blvd, CA 95817, USA; 2 Department of Medical Pathology and Laboratory Medicine, University of California at Davis, Davis, CA 95817, USA; 3 Department of Neurology, Programs in Neuroscience, Developmental Biology and Regenerative Medicine, University of California at San Francisco, 1550 4th Street, CA 94158, USA

## Abstract

**Background:**

Tangential migration presents the primary mode of migration of cortical interneurons translocating into the cerebral cortex from subpallial domains. This migration takes place in multiple streams with the most superficial one located in the cortical marginal zone. While a number of forebrain-expressed molecules regulating this process have emerged, it remains unclear to what extent structures outside the brain, like the forebrain meninges, are involved.

**Results:**

We studied a unique *Foxc1 *hypomorph mouse model (*Foxc1^hith/hith^*) with meningeal defects and impaired tangential migration of cortical interneurons. We identified a territorial correlation between meningeal defects and disruption of interneuron migration along the adjacent marginal zone in these animals, suggesting that impaired meningeal integrity might be the primary cause for the observed migration defects. Moreover, we postulate that the meningeal factor regulating tangential migration that is affected in homozygote mutants is the chemokine Cxcl12. In addition, by using chromatin immunoprecipitation analysis, we provide evidence that the *Cxcl12 *gene is a direct transcriptional target of Foxc1 in the meninges. Further, we observe migration defects of a lesser degree in Cajal-Retzius cells migrating within the cortical marginal zone, indicating a less important role for Cxcl12 in their migration. Finally, the developmental migration defects observed in *Foxc1^hith/hith ^*mutants do not lead to obvious differences in interneuron distribution in the adult if compared to control animals.

**Conclusions:**

Our results suggest a critical role for the forebrain meninges to promote during development the tangential migration of cortical interneurons along the cortical marginal zone and Cxcl12 as the factor responsible for this property.

## Background

The cerebral cortex's proper functioning depends on the balance between excitatory projection neurons and inhibitory interneurons. In rodents, most GABA (γ-aminobutyric acid)-producing interneurons of the cerebral cortex originate in the medial ganglionic eminence of the ventral forebrain and migrate to their cortical destinations using a tangential route [1,2]. The subcortical origin and complex migratory path of cortical interneurons differ greatly from the origin of the cortical projection neurons and their radial migratory route. Once interneurons reach the cortex they follow mostly stereotypical routes in the marginal zone (MZ) and the subventricular zone (SVZ)/intermediate zone (IZ). Upon reaching their eventual dorsoventral position within the cortical sheath, interneurons migrate radially to adjust for laminar positioning. Several factors that regulate tangential migration during development have been identified, including ones expressed by the brain and/or by the meninges [3-6]. Despite the recently identified role of meningeally produced chemoattractants in regulating migration, many details as to how the meninges control tangential migration remain unresolved. To specifically address the role of the meninges, we examined interneuron migration in mice with defective meningeal development caused by a point mutation in *Foxc1 *(forkhead box c1) [7]. This novel allele (*Foxc1^hith^*) represents a hypomorph resulting from protein destabilization. We have previously demonstrated the central role of Foxc1 in the development of the meninges and provided insights into the role of the meninges in controlling the development of adjacent structures - the skull and cerebral cortex [8,9]. In this paper, we show that proper meningeal function is required for guidance of cortical interneurons along the cortical MZ during their tangential migration. Developmentally, we observed that *Foxc1^hith/hith ^*mice show reduced migration in the most dorsal aspects of the cortex at the peak of tangential migration (embryonic day (E)14.5 to E18.5). Since this reduction in interneuron precursors affects only the superficial migratory stream, within the MZ, and not the deeper migratory stream in the intermediate zone, we examined expression of a regulator of tangential migration in the MZ, the chemokine Cxcl12 (chemokine (C-X-C motif) ligand 12; Sdf1). We found that Cxcl12 expression in the MZ and overlying leptomeninges is severely reduced in mutant mice, implying its regulation by meningeally expressed Foxc1. Indeed, using chromatin immunoprecipitation (ChIP) analysis to identify Foxc1 complexes bound to *Cxcl12 *regulatory sequences, we were able to confirm such complexes and consequently the *Cxcl12 *gene as a direct target of Foxc1 transcriptional activity. Residual *Cxcl12 *expression in the dorsal forebrain is entirely derived from Cajal-Retzius cells (CRCs), whose tangential migration is more mildly affected in *Foxc1^hith/hith ^*mice.

## Results

### Tangential migration defects in *Foxc1^hith/hith ^*fetuses

The *hith *allele was recovered in a forward genetic screen using ethyl-nitroso-urea (ENU) mutagenesis [7] and was characterized as a hypomorph of *Foxc1 *(F107L) [8]. The screen, which led to the identification of this line of mice, was specifically designed to identify mutations disrupting forebrain development. This aim was facilitated by the use of mice carrying the *Dlx5/6-lacZ *transgene [10], which allows for the expression of β-galactosidase (β-gal) in the forebrain expression domains of the *Dlx5/6 *genes. The expression domains of these genes include the ganglionic eminences of the basal telencephalon and the GABA-ergic interneuron precursors, cells that originate in the ganglionic eminences and subsequently migrate tangentially to subcortical domains and the developing cerebral cortex.

**Figure 1:**
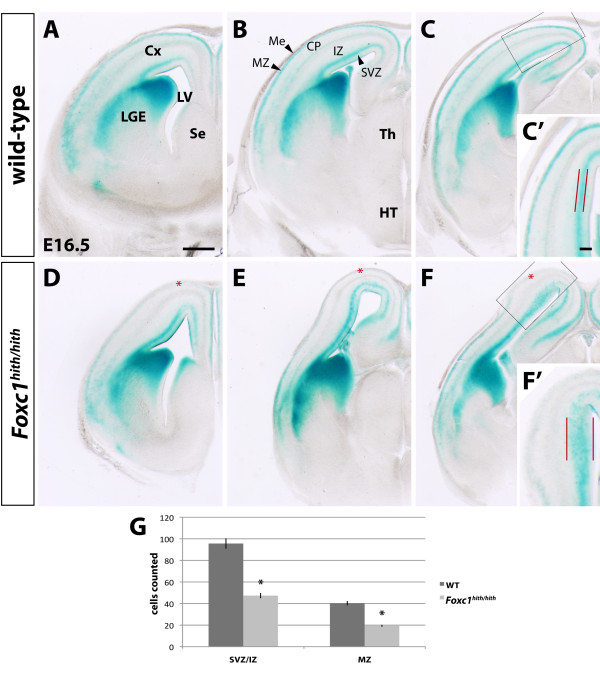
**Cortical interneuron migration defects of *Foxc1^hith/hith ^*fetuses**. **(A-F) **Coronal hemi-sections through the X-gal stained forebrains of wild-type (WT) (A-C) and *Foxc1^hith/hith ^*(D-F) fetuses expressing the *Dlx5/6*-*lacZ *transgene at E16.5. From left to right, three, progressively more posterior levels through the forebrain are shown. Red asterisks in (D-F) indicate the disruption of interneuron migration in the mutant marginal zone (MZ). The close-ups in (C', F') show the increased width of the deep migratory stream, at the intersection of the subventricular zone with the intemediate zone (SVZ/IZ), in the dorsal cortex of the mutant. **(G) **The results of the quantitative analysis of Dlx5/6+ migrating interneurons for the SVZ/IZ and MZ, respectively. Error bars indicate average deviation. The cell counts were conducted on a 500 μm long cortical segment of the dorsal cortex comparable to the close ups in (C', F'). In the mutant the number of labeled cells is significantly decreased in both migratory streams (SVZ/IZ, *P *= 0.019; MZ, *P *= 0.024). Asterisks indicate statistical significance. Scale bars: 500 μm (A-F); 100 μm (C', F'). CP, cortical plate; Cx, cerebral cortex; HT, hypothalamus; IZ, intermediate zone; LGE, lateral ganglionic eminence; LV, lateral ventricle; Me, meninges; MZ, marginal zone; Se, septum; SVZ, subventricular zone; Th, thalamus.

Comparing homozygous *hith *mutants to wild-type (WT) littermates at several developmental stages (E14.5 to E17.5) we identified a consistent difference in the β-gal expression pattern with respect to labeled interneurons migrating along the MZ (heterozygous mice were phenotypically indistinguishable from WT mice). In the mutants, the staining intensity was significantly decreased or absent in superficial aspects of the dorsal developing cortex, indicating a reduction in β-gal+ cells following this migratory route (Figure 1 and data not shown). In contrast, the migratory path of interneurons following the SVZ/IZ was not affected laterally and widened dorsally (Figure 1C, C', F, F'). We went on to quantify the number of β-gal+ cells within the two migratory streams in a 500 μm long segment of the dorsal cortex and found them to be significantly reduced in affected mutants for either stream (WT SVZ/IZ 96 ± 16 (mean ± average deviation), WT MZ 40 ± 7, mutant SVZ/IZ 47 ± 8, mutant MZ 19 ± 2, Student's *t*-test, SVZ/IZ *P *= 0.019, MZ *P *= 0.024, n = 3 for either genotype). Interestingly, the MZ of the cortical medial wall shows considerable staining for the *Dlx5/6 *transgene comparable to that in the WT (Figure 1), indicative of the less severe meningeal defects in this region compared to the dorsolateral cortical meninges [8]. Staining in all other β-gal+ positive structures, like the ganglionic eminences, was unaffected, implying a very specific defect restricted to this localized population of cells. The reduction in staining in more superficial regions of the MZ parallels the meningeal defects, which have been previously characterized in this line of mice and are also most severe in the dorsolateral cortex [8]. This territorial correlation between meningeal and interneuron phenotype indicates that disrupted meningeal integrity might be the primary cause for the observed defects in interneuron migration along the adjacent MZ. This explanation is appealing compared to the idea of a cell-autonomous defect in cortical interneurons considering that *Foxc1*, the mutated gene in *hith *mice, is expressed in the meninges but not in cortical interneurons [8,11].

### Foxc1 directly regulates *Cxcl12 *expression in the meninges

**Figure 2:**
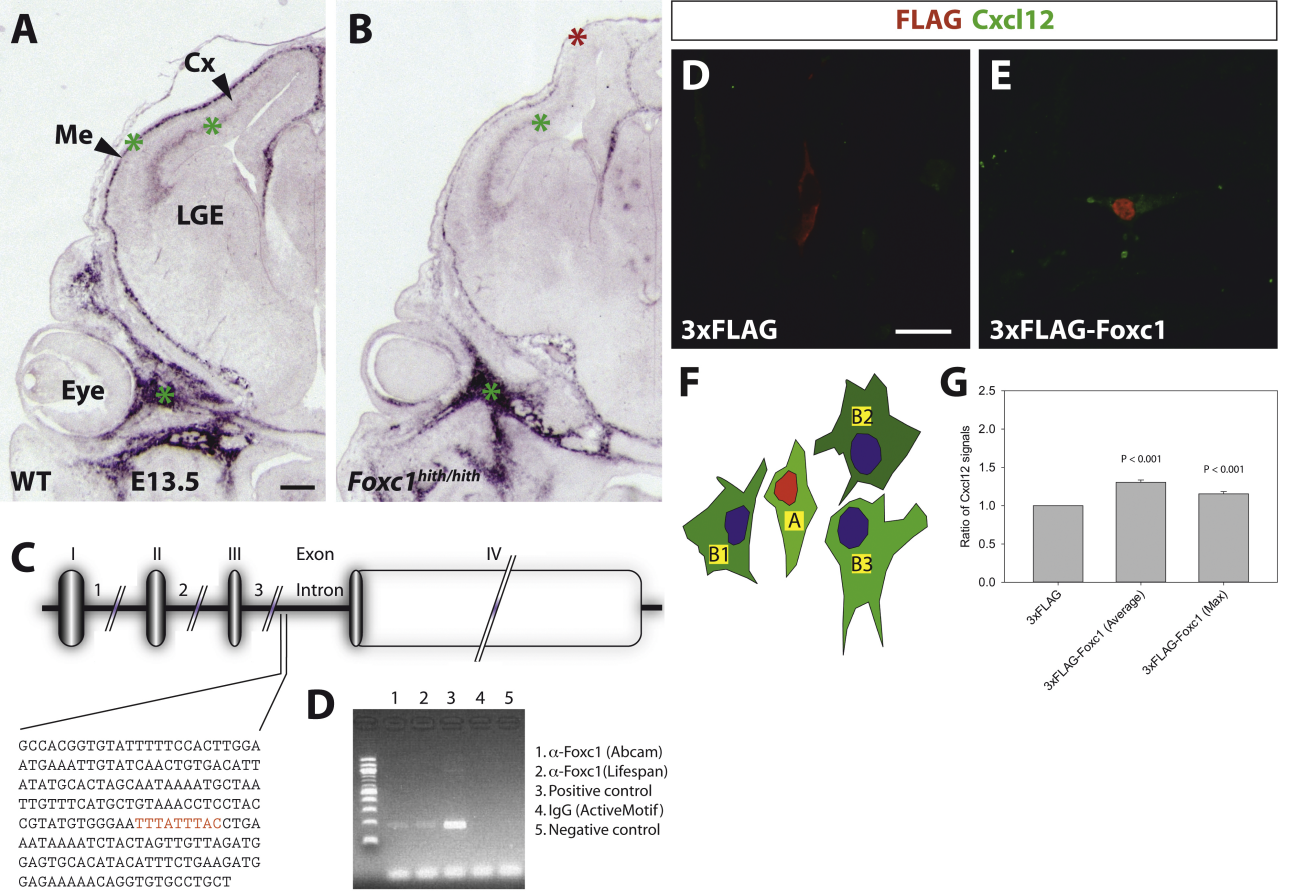
**Meningeal *Cxcl12 *expression is reduced in *Foxc1^hith/hith ^*mutants and directly regulated by Foxc1**. **(A, B) ***Cxcl12 *expression analysis on coronal sections of wild-type (WT) and *Foxc1^hith/hith ^*embryos at E13.5 shows transcripts in the meninges, cortical SVZ/IZ, and periocular mesenchyme (green asterisks). Meningeal *Cxcl12 *expression is reduced or absent (red asterisk in (B)) throughout the forebrain meninges of the mutant embryo while expression in the cortical SVZ/IZ and facial mesenchyme is not affected (green asterisks in (B)). **(C) **A scheme of the *Cxcl12 *gene with exons shown as boxes and the coding region darkened. The sequence of the fragment amplified in the ChIP analysis is shown below with the reverse complement of the Foxc1 binding consensus sequence GTAAATAAA highlighted in red. **(D) **The results of the ChIP analysis where in lanes 1 and 2 the fragment containing the Foxc1 binding site was amplified from DNA templates pulled down with two different Foxc1 antibodies. Genomic DNA was used in the positive control (lane 3), DNA pulled down with an IgG antibody in the negative control (lane 4), and no template DNA in lane 5. **(E, F) **Transfection of meningeal fibroblasts in primary culture with an expression construct for Foxc1 (F) and control plasmid (E). **(G) **Illustration of the parameters used to quantify the average ratio of Cxcl12 immunofluorescence signal intensity [3xFLAG-Foxc1 (Average) = A/(B1 + B2 + B3)/3] or maximum ratio of signal intensity [3xFLAG-Foxc1 (Max) = A/Max (B1 + B2+ B3)]. A, Foxc1-transfected cell; B1 to B3, neighboring not transfected cells. **(H) **The results of the analysis and significant upregulation of Cxcl12 in 3xFLAG-Foxc1-transfected cells. Error bars indicate the average deviation. Scale bars: 200 μm (A, B); 20 μm (D, E). Cx, cerebral cortex; LGE, lateral ganglionic eminence; Me, meninges.

**Figure 3:**
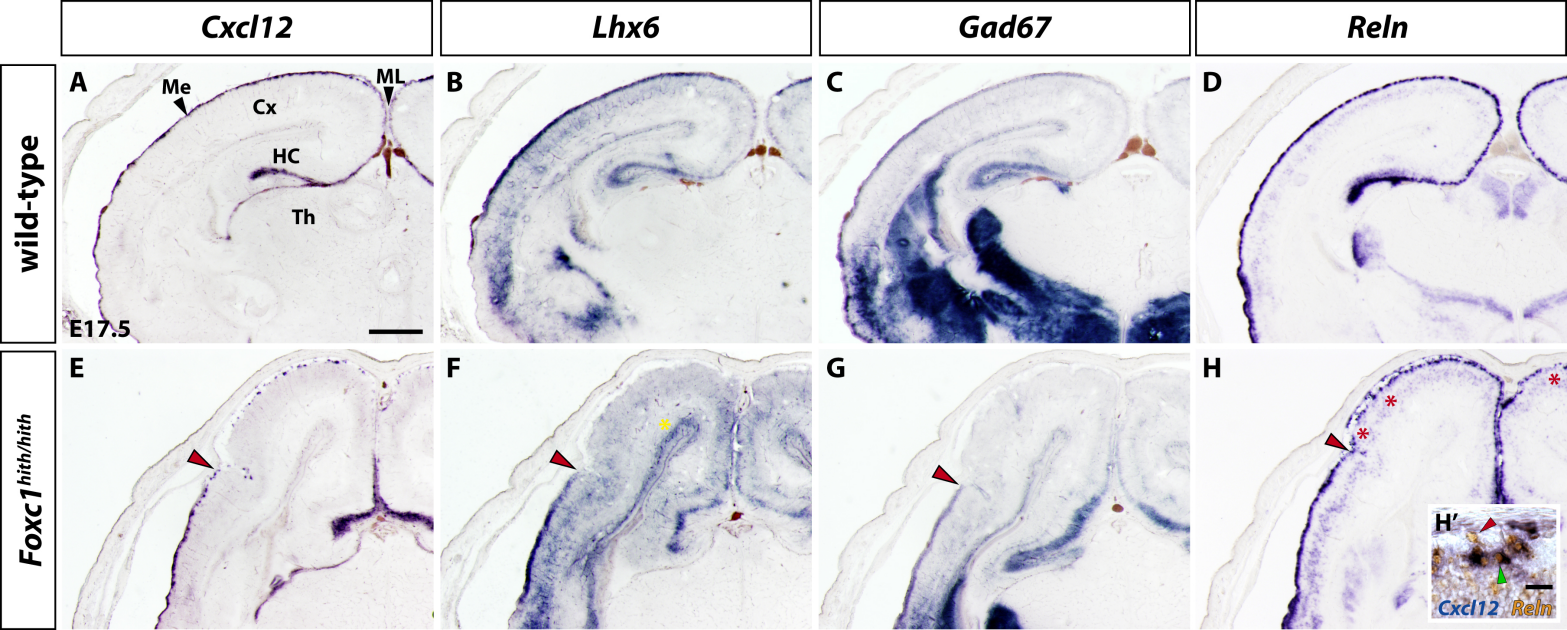
**Distribution of tangentially migrating cells analyzed by RNA *in situ *hybridization**. **(A-H) **Coronal sections of WT (A-D) and *Foxc1^hith/hith ^*(E-H) embryos at E17.5. Expression of *Cxcl12 *in the mutant appears fragmented in dorsal aspects and unaffected in ventrolateral aspects of the meninges (E). Expression of cortical interneuron markers *Lhx6 *and *Gad67*, although continuous in the WT (B, C), is absent in dorsal aspects of the cortical marginal zone in the mutant (G, H). The loss of expression of these two markers in the MZ starts exactly at the level where *Cxcl12 *expression breaks down as indicated by red arrowheads. Interestingly, expression of *Lhx6 *is increased and widened in the dorsal SVZ of the mutant (F, yellow asterisk), consistent with the β-gal expression results from Figure 1. Expression of *Reln*, which marks CRCs appears continuous in the WT (D) but is mildly disrupted in the dorsal aspect of the mutant (H). The breakup of continuous *Reln *expression occurs at the same lateral position as *Cxcl12 *disruption (red arrowhead) but appears less fragmented than *Cxcl12 *expression. Gaps in *Reln *expression in the MZ are indicated by red asterisks (H). A close-up of the dorsal cortex in a homozygote mutant shows double RNA *in situ *hybridization analysis for *Cxcl12 *and *Reln *(H'), confirming the expression of *Cxcl12 *by CRCs. All *Cxcl12*+ cells are also positive for the expression of *Reln*. Note also that some *Reln+ *cells do not express *Cxcl12 *(red arrowhead). Scale bars: 200 μm (A-H); 20 μm (H'). Cx, cerebral cortex; HC, hippocampus; Me, meninges; ML, midline; Th, thalamus.

Recent studies have shown that the meninges produce chemical cues regulating cell migration during cortical development. Cxcl12, a chemokine ligand produced by the innermost layer of the meninges, the pia mater, regulates the positioning of cortical interneurons and CRCs by acting as a chemoattractant for these cells during embryonic and fetal life [6,12-14]. In addition, Cxcl12 expression in the cortical SVZ/IZ is essential for recognition of this pathway by interneurons, with animal models demonstrating migration defects in the absence of Cxcl12 signaling [15]. Both interneuron and CRC response to meningeal Cxcl12 is mediated by the Cxcr4 receptor (C-X-C chemokine receptor 4). These findings prompted us to examine the expression of *Cxcl12 *in the *hith/hith *mouse using RNA *in situ *hybridization at different developmental stages ranging from E12.5 to E18.5. Our expression analysis matched previous findings on the regional distribution of *Cxcl12 *[16] with relevant expression seen in the pia mater and SVZ/IZ (Figure 2 and data not shown). Interestingly, upon examining *hith/hith *mutants, we saw changes in expression levels and distribution of *Cxcl12 *from early on compared to littermate controls. At E13.5, *Cxcl12 *expression in the forebrain meninges is reduced laterally and absent dorsally (Figure 2B). This remarkable reduction of *Cxcl12 *expression in the meninges is not accompanied by any reduced RNA levels in other analyzed expression domains, either within the nervous system (the cortical SVZ/IZ) or outside the brain (the facial mesenchyme). Later in development, at E17.5, basolateral aspects of the forebrain meninges show no differences in expression levels of *Cxcl12 *but dorsal to lateral levels display significant disruption in the continuity of *Cxcl12 *expression (Figure 3E).

The reduction in *Cxcl12 *expression seen in homozygous *hith *mutants raises the question of whether expression of *Cxcl12 *in the meninges is directly regulated by Foxc1. To address this question, we performed chromatin immunoprecipitation (ChIP) assays with Foxc1 immunopurified lysates from meningeal tissue aimed at amplifying *Cxcl12 *sequences containing Foxc1 binding sites (Figure 2C, D). Using this approach we amplified a fragment from intron 3 of the *Cxcl12 *gene, which contains a Foxc1 binding site (GTAAATAAA) and thereby identified the *Cxcl12 *gene as a direct target of Foxc1 transcriptional activity. To further strengthen the evidence for a direct regulation of *Cxcl12 *by Foxc1, we overexpressed Foxc1 in cultured primary meningeal cells by transfecting them with FLAG-tagged Foxc1-expression constructs and analyzed potential Cxcl12 upregulation by immunofluorescent detection. To quantify the ratio of Cxcl12 immunofluorescence in transfected over not transfected cells, we established the average ratio of signal intensity [3xFLAG-Foxc1 (Average) = A/(B1 + B2 + B3)/3] and maximum ratio of signal intensity [3xFLAG-Foxc1 (Max) = A/Max (B1 + B2+ B3)/3]. Our results confirmed a significant upregulation of Cxcl12 in Foxc1 transfected cells over not transfected cells (Student's *t*-test, average ratio *P *< 0.001, maximum ratio *P *< 0.001; Figure 2D-G).

### Interneuron distribution in the marginal zone matches *Cxcl12 *expression

To map interneuron distribution at E17.5, we analyzed *Gad67 *(*Gad1*) and *Lhx6 *expression, two markers of cortical interneuron identity, in sections adjacent to those analyzed for *Cxcl12 *expression. In both cases we saw robust expression in the cortical MZ at basolateral aspects, indicating the presence of interneurons (Figure 3F, G). At dorsal aspects, however, expression is absent, with the interruption occurring at exactly the same lateral position as for *Cxcl12*, suggesting a close relationship between *Cxcl12 *expression and the presence of MZ interneuron precursors (Figure 3F, G). This finding strongly suggests that meningeal integrity is necessary for proper guidance of interneurons during their tangential migration along the adjacent cortical MZ. In addition, impaired expression of Cxcl12 is likely causative of the observed phenotype since several studies have demonstrated the important role Cxcl12 plays as a chemoattractant for both interneurons and CRCs.

During development, CRCs are also distributed over the cortical surface through tangential migration within the MZ and recent studies point also here to Cxcl12 as one of the chemoattractive signals mediating their movement across the cortical surface [12,13]. In order to visualize CRCs in homozygous mutants, we used *Reelin *(*Reln*) RNA *in situ *hybridization. We saw *Reln *expression in the mutants showing a similar pattern of disruption in dorsomedial aspects of the cortex, as it is the case for *Cxcl12*, suggesting migration defects in CRCs as well. Specifically, dorsolateral positions characterized by discontinuous *Cxcl12 *expression show also slightly disrupted, though more robust, *Reln *expression with occasional gaps in *Reln*+ cells (Figure 3H). Since CRCs themselves express *Cxcl12 *at later developmental stages and might be the source of *Cxcl12 *expression seen at E17.5 in *hith/hith *mutants, we decided to explore this possibility. Using double *in situ *hybridization analysis with *Cxcl12 *and *Reln *probes, we identified all *Cxcl12 *expression in the dorsal cortex as being derived from CRCs and of non-meningeal origin (Figure 3H').

### Distribution of cortical interneurons in postnatal stages

**Figure 4:**
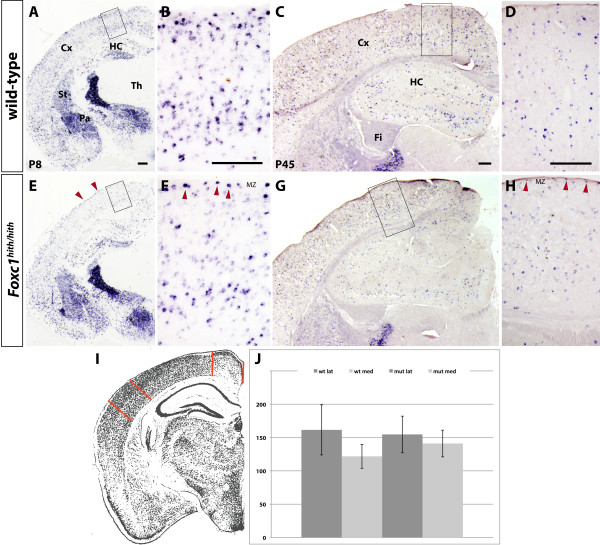
**Analysis of postnatal distribution of cortical interneurons**. **(A-H) ***Gad67 in situ *hybridization analysis is shown on coronal hemi-sections through the forebrains of postnatal day (P)8 (A, E) and adult animals (C, G); in (B, F, D, H) sections of the cortices of the P8 and P45 brains, respectively, are shown in higher magnification. The regions shown in greater detail in the close-ups are outlined in the hemisections. *Gad67+ *interneurons can be seen throughout the entire width of the mutant cortex without exclusion from superficial layers both at P8 and in the adult (red arrowheads). The distribution of interneurons in the adult cortex was quantified by counting the cells in a 1 mm segment from lateral and medial cortex, respectively. **(I) **Schematic of a forebrain hemi-section illustrates the positions (framed by red bars) of the cortical segments that were counted. **(J) **Numbers of Gad67+ cells counted for WT and mutant medial (med) and lateral (lat) positions. Error bars indicate standard error. No significant differences in interneuron distribution were observed for the positions analyzed. All scale bars represent 250 μm. Cx, cerebral cortex; Fi, fimbria; HC, hippocampus; MZ, marginal zone; Pa, pallidum; St, striatum; Th, thalamus.

To gain insight into the consequences of the aberrant interneuron migration in the adult cortex, we examined the distribution of cortical interneurons in *hith/hith *mice at postnatal stages. Using *Gad67 in situ *hybridization to label interneurons, we did not see tangible differences in their laminar distribution at P8 or in the adult brain. Specifically, at P8, we saw a great number of labeled cells present in the MZ (Figure 4E, F). In the adult (P45), *Gad67*+ cells can be seen throughout the entire extent of the cortical MZ and upper cortical layers pointing towards corrective mechanisms in the postnatal distribution and positioning of cortical interneurons (Figure 4). We went on to quantify the number of interneurons present in two different segments of the adult cerebral cortex. For our analysis, we chose a bregma position of approximately -1.8, which corresponds to the position of severe migration defects at embryonic stages and we analyzed a lateral (somatosensory cortex) and a medial area (retrosplenial cortex). The lateral position corresponds to an area of unaffected MZ interneuron migration as identified in embryonic stages while the medial position corresponds to an area of maximal migration defects developmentally. Total count of interneurons in a 1 mm cortical segment revealed no significant differences for the two positions between homozygote mutants (n = 5) and WT (n = 4) animals (WT lateral 162 ± 38 (mean ± standard error), WT medial 122 ± 18, mutant lateral 155 ± 28, mutant medial 141 ± 20, Mann-Whitney test, medial *P *= 0.555, lateral *P *= 0.905). This result suggests no actual loss of interneurons in the dorsolateral cortex of affected animals despite the severity of embryonic migration defects for this cortical segment.

**Figure 5:**
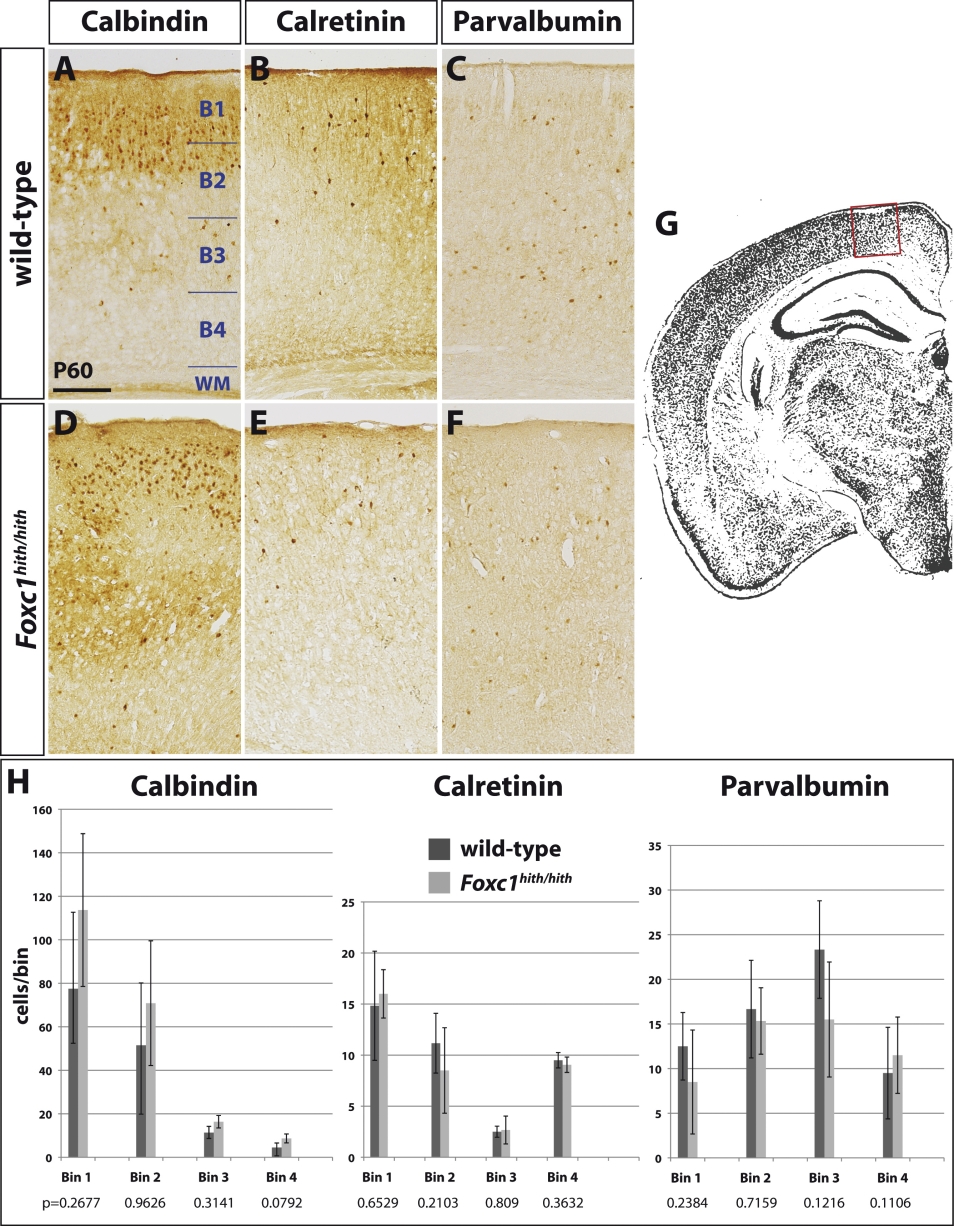
**Postnatal laminar distribution of cortical interneuron subtypes**. **(A-F) **Immunohistochemical detection of interneuron subtypes carried out for calbindin (A, D), calretinin (B, E), and parvalbumin (C, F) positive cells in the adult cortex. **(G) **The analysis was carried out on a cortical segment of 400 μm length as shown. Cortical sections were divided between the marginal zone and white matter (WM) in four horizontal bins (B1 to B4) and immunolabeled cells of each bin counted. Scale bar: 100 μm. **(H) **Quantification and laminar comparison between WT and *Foxc1^hith/hith ^*mutants. Error bars indicate standard error. No significant differences in the laminar distribution of interneuron subtypes were observed and the *P*-values for each pairwise comparison is indicated below the bar diagrams.

To investigate further the distribution of interneurons in the adult cortex of *hith/hith *mice, we analyzed the laminar distribution of three interneuron subtypes characterized by the expression of the neuropeptides calbindin, calretinin, and parvalbumin. To that effect, we performed immunohistocehmistry for the detection of calbindin-, calretinin-, and parvalbumin-positive cells on sections of adult brains and counted cells in four laminar bins equally divided between the pial surface and the white matter (Figure 5). For our analysis, we counted cells within a cortical segment of 400 μm length at bregma position -1.8 and at a midpoint distance of 800 μm form the cortical midline for either genotype. This position correlates developmentally with the most severe defects in interneuron migration along the MZ. Comparing the numbers of interneurons for every bin between mutant and WT, we did not identify any significant differences in the laminar distribution of interneuron subtypes in the adult cortex of affected animals (Figure 5H).

## Discussion

In this study, we provide evidence that the pallial meninges regulate tangential migration of interneuron precursors. In mice homozygous for a *Foxc1 *mutation (*hith*) meningeal integrity and function is impaired, which has profound, negative effects on the ability of GABA-ergic interneurons to migrate along the adjacent cortical MZ. The migrating interneurons are absent from the dorsolateral cortex, which raises the question as to where these cells accumulate and/or migrate. Careful analysis of cortical interneuron distribution in the developing brain using the *Dlx5/6-lacZ *transgenic marker and *Gad67 *and *Lhx6 in situ *hybridization analysis failed to show any region of increased presence of labeled cells. Interestingly, the most dorsal aspect of the SVZ/IZ migratory stream of homozygous mutants appears thicker as a result of a more loose packing of migrating interneurons. This finding may parallel the developmental lamination defects observed in mice deficient for the Cxcl12 receptors Cxcr4 and Cxcr7, which are also characterized by a less dense packing of the streams of migrating intereneurons [17,18]. The appearance of the migration defects in the *Foxc1^hith/hith ^*mutants likely also precludes alternative explanations for their causes, like defects centered on the pallial-subpallial boundary, an important signaling center regulating tangential migration. In such an instance, we would expect a more global phenotype affecting tangential migration of medial ganglionic eminence-generated interneurons in all cortical aspects and not only the dorsolateral MZ, which correlates with both *Cxcl12 *downregulation and meningeal defects in this area.

Our data, in conjunction with other work, indicate that the meningeally expressed factor guiding tangential migration along the MZ is very likely Cxcl12. We show that meningeal *Cxcl12 *expression is severely decreased or entirely absent in *Foxc1^hith/hith ^*mutants during the period of tangential interneuron migration. At E13.5 *Cxcl12 *expression is reduced throughout the meninges while at E17.5 basolateral aspects show intact expression levels but dorsal aspects show reduced ones, which might point towards a developmental delay in *Cxcl12 *expression of the basolateral meninges. Support for the hypothesis that Cxcl12 is the chemo-attractant factor for MZ interneurons comes from several recent studies. For instance, it has been shown that Tbr2+ cells of the cortical SVZ express Cxcl12 and forced expression of *Tbr2 *by *in vivo *electroporation leads to concomitant *Cxcl12 *upregulation associated with interneuron accumulating foci at the electroporation sites [19]. Further, Cxcl12 signaling in responsive cells is initiated upon binding to the chemokine receptors Cxcr4 and Cxcr7 and both receptors are coexpressed in migrating interneurons. Interestingly, inactivation of either receptor leads to comparable interneuron migration defects due to their distinct functions in these cells [17,18]. While both receptors bind Cxcl12, Cxcr7 is internalized upon ligand binding and essential for the modulation of Cxcr4 function by regulating Cxcr4 protein levels at the cell surface [18]. Thereby by controlling the amount of chemokine receptor protein present, Cxcr7 modulates chemokine responsiveness in migrating interneurons. In addition, it has been shown that meningeally expressed Cxcl12 directs migrating neurons not only in the forebrain but also in the cerebellum. Here, it has been demonstrated that neuronal precursors of the external germinal layer are attracted by meningeally expressed Cxcl12, which regulates their positioning in the external granule cell layer until they differentiate and lose Cxcl12 responsiveness [20]. Thus, the attractive interaction between Cxcl12-expressing meningeal cells and the Cxcr4-expressing neurons appears to be a conserved and reappearing mode in the regulation of neuronal migration.

We considered the idea that expression of *Cxcl12 *in the meninges might be directly regulated by Foxc1 and tested this hypothesis through ChIP analysis. We identified a Foxc1 consensus-binding site within the third intron of the *Cxcl12 *gene and verified complex formation between Foxc1 and this fragment. In addition, forced overexpression of Foxc1 in transfected meningeal cells leads to a significant upregulation of Cxcl12. This analysis allowed us to add the *Cxcl12 *gene to the list of direct targets of Foxc1 transcriptional activity and, to our knowledge, the first one expressed in the meninges. Interestingly, although Foxc1 and *Cxcl12 *expression also overlap in the facial mesenchyme, we do not see any downregulation of *Cxcl12 *in the facial mesenchyme.

CRCs in the dorsal MZ, although also dependent on Cxcl12 signaling for their proper migration, were considerably less disturbed in their positioning than interneurons in the *Foxc1^hith/hith ^*mutants. This finding, in combination with the fact that Foxc1 directly activates meningeal *Cxcl12 *transcription, points towards tangential migration defects being a primary defect rather than the consequence of a disrupted trophic role of the meninges leading to more generic defects in corticogenesis and CRCs and/or interneuron survival as previously reported [21]. CRCs that originate in the cortical hem express the Cxcl12 receptor Cxcr4 and in Cxcr4-deficient mice a fraction of CRCs becomes ectopically positioned by leaving the MZ and invading the cortical plate [12]. The fact that, despite reduced Cxcl12 signaling from the meninges in *Foxc1^hith/hith ^*mice, we do not see any major disruption of CRC distribution might be best explained by the hypomorphic nature of the *hith *allele and incomplete loss of Cxcl12 signaling in the meninges early in development. There is also the possibility that additional factors regulate CRC positioning in the MZ as there are three focal sources of CRCs that have been identified in mice - the cortical hem, the ventral pallium, and the septum - and Cxcl12 has been demonstrated to influence only the migration of hem-derived CRCs [12]. The subpopulations derived from the ventral pallium and septum do not express Cxcr4, and are therefore largely insensitive to Cxcl12.

Surprisingly, developmental migration defects in interneurons of *Foxc1^hith/hith ^*mutants do not lead to obvious differences in adult distribution compared to the WT, although this later finding remains difficult to evaluate in great detail considering the overlying massive cortical dysplasia seen in the mutants. However, we undertook a quantitative evaluation of the distribution of interneurons by comparing two aspects of the adult cortex. A medial aspect corresponding to the most severe developmental migration defects and a lateral aspect without obvious migration defects during development. Confirming our initial impression that neither medial nor lateral cortical aspects show any significant differences between homozygote mutants and WT in the number of interneurons present. In addition, the laminar distribution of interneuron subtypes, positive for the expression of calbindin, calretinin, and parvalbumin, is also not affected in the adult cortex of *hith/hith *mice, suggesting a corrective mechanism in postnatal development, which guides interneurons to their correct location. This result is also consistent with recent work showing that the laminar interneuron distribution in the postnatal cortex is almost completely restored in Tbr2 mutants lacking developmentally the deep stream of tangential interneuron migration along the SVZ/IZ [19].

The fact that at postnatal stages no obvious differences can be seen in the distribution of cortical interneurons of *Foxc1^hith/hith ^*mice compared to the WT may be best explained by the movements these cells undertake later in development. While their initial route of migration is tangential, once interneurons reach their approximate lateral position within the pallium they undergo extensive radial migration [22-25]. These cell movements, perpendicular to the overall tangential mode of migration, can be directed both from the MZ to the ventricular surface and from the intermediate zone to the pial surface. Apparently, these radial movements of GABA-ergic interneurons provide for correct layer positioning and proper integration into the cortical circuitry and likely present the source of cells compensating for the observed deficiency in interneurons in the MZ of *Foxc1^hith/hith ^*mice. In addition, it appears likely that layer positioning of interneurons perinatally occurs independently of Cxcl12, since recent work in our laboratory demonstrated a developmental loss of interneuron response to Cxcl12 signaling [6]. Such a postnatal loss of responsiveness to meningeally expressed Cxcl12 has been also shown for cerebellar granule cells as they differentiate and radially migrate through the Purkinje cell layer to the internal granule cell layer [20].

## Conclusions

Our study, through the analysis of a *Foxc1 *hypomorphic mouse model, suggests a critical developmental role for the dorsal forebrain meninges to promote tangential migration of cortical interneurons in the adjacent MZ. The molecule mediating this property appears to be Cxcl12, a chemokine whose expression we show to be directly regulated by Foxc1.

## Materials and methods

### Animal husbandry and genotyping

Mice are housed in specific-pathogen-free facilities approved by AALAC. All animals were handled in accordance with protocols approved by the UCSF and UC Davis Committees on Animal Research. The colony of animals carrying the *Foxc1^hith ^*allele (induced on C57BL/6J background) is maintained by crossing male carriers with FVB/NJ females carrying the *Dlx5/6-lacZ *transgene in a homozygous state. This mode of outcross is currently in the sixth generation without any changes in penetrance or variability of the mutant phenotype. Genotyping was performed as previously described [8].

### X-gal staining

Staining for β-gal activity indicating *Dlx5/6-lacZ *expression was performed using X-gal as a substrate on whole-mount heads or embryos according to standard protocols and as previously described [7]. Subsequently, using a vibrating microtome, 100 μm thick sections were cut, mounted on slides, and photographed. For the quantification of individual cells, stained brains were cryoprotectively embedded and sectioned at 20 μm on a freezing microtome. A dorsolateral cortical segment of 500 μm length just below the apex was selected for the cell counts.

### RNA *in situ *hybridization

*In situ *hybridizations using digoxigenin-labeled antisense RNA probes were carried out following standard protocols and as previously described [26]. Double *in situ *hybridizations using digoxigenin- and fluorescein-labeled antisense RNA probes were carried out following standard protocols. *In vitro *transcriptions to generate labeled riboprobes were performed on plasmids obtained from Open Biosystems (*Cxcl12*, IMAGE clone 3984385; *Reln*, IMAGE clone 734262) and John LR Rubenstein (*Gad67 *[27]; *Lhx6 *[28]).

### Immunohistochemistry

Frozen tissue was sectioned at 14 μm and processed for immunostaining using standard protocols. Antibodies used were calbindin (1:5,000, Swant, Marly, Friborurg, Switzerland), calretinin (1:2,000, Chemicon/Millipore, Billerica, Massachusetts, USA) parvalbumin (Swant, 1:5,000).

### Chromatin immunoprecipitation

Meningeal tissue was collected from E16.5 CD1 fetuses in Hank's balanced salt solution. The solution was changed to the ice-cold lysis buffer and applied to an ice-cold dounce homogenizer (Bellco, Vineland, New Jersey, USA) for ten strokes to release the nuclei. For chromatin shearing we used a Branson Sonifier 450 for ten pulses of 12 seconds each with a 1 minute rest on ice between each pulse at 37% power. The lysate was incubated with α-Foxc1 polyclonal antibodies (Abcam, Cambridge, Massachusetts, USA) to which 20 μl of sheep anti-rabbit IgG dynabeads (Invitrogen/Life technologies, Grand Island, New York, USA) were added to immunoprecipitate any complexes formed. For PCR amplification of Cxcl12 genomic fragments from the immunoprecipitated chromatins, we used the following DNA primers: Cxcl12_197u (5'-GCCACGGTGTATTTTTCCAC-3') and Cxcl12_197d (5'-AGCAGGCACACCTGTTTTTC-3'). The target sequence for these primers lies on intron 3 of *Cxcl12 *(NM_001012477.1), which contains the previously identified GTAAATAAA Foxc1 consensus binding site [29].

### Culture and transfection of meningeal cells

Meningeal cells were collected from three litters of E16.5 CD1 embryos and grown for 24 h. 3xFLAG-tagged Foxc1 and control expression vectors were transfected using Lipofectamine plus reagent (Invitrogen) for 40 h. After transfection cells were fixed in 4% paraformaldehyde in phosphate-buffered saline and processed for immunostaining. Cells were pretreated with boiling hot citric acid (pH 6.0) for antigen retrieval and immunostained for Cxcl12 (1:100, eBioscience, San Diego, California, USA). To measure the intensity of Cxcl12 signals, we used the ratio of the signals of transfected cells versus non-transfected neighboring cells. The experiment was repeated twice and data were collected from 66 transfected cells.

## Abbreviations

ChIP: chromatin immunoprecipitation; CRC: Cajal-Retzius cell; E: embryonic day; GABA: γ-aminobutyric acid; gal: galactosidase; IZ: intermediate zone; MZ: marginal zone; SVZ: subventricular zone; WT: wild type.

## Competing interests

The authors declare that they have no competing interests.

## Authors' contributions

KZ designed and carried out most of the experiments and co-wrote the paper. YC, JAS, and LAO designed and carried out some of the experiments and co-wrote the paper. SJP participated in design, supervision, and analysis and co-wrote the paper. All authors read and approved the final manuscript.
